# Computer Vision Positioning and Local Obstacle Avoidance Optimization Based on Neural Network Algorithm

**DOI:** 10.1155/2022/3061910

**Published:** 2022-04-01

**Authors:** Lei Yang, Weimin Lei

**Affiliations:** ^1^School of Computer Science and Engineering, Northeastern University, Shenyang 110169, China; ^2^Engineering Research Center of Security Technology of Complex Network System, Ministry of Education, Beijing 100000, China

## Abstract

Due to the rapid development of social computerization and smart devices, there is an increasing demand for indoor positioning of mobile robots in the robotics field, so it is very important to realize the autonomous navigation of mobile robots. However, in indoor scenes, due to factors such as dark walls, the global positioning system cannot effectively locate, and the broadband and wired positioning technologies used indoors have problems such as base station laying and delay. Computer vision positioning technology has greatly improved the camera hardware due to its simple equipment and low cost. Compared with other sensor cameras, it is less affected by environmental changes, so visual positioning has received extensive attention. Image matching has become the most critical link in visual positioning. The accuracy, speed, and robustness of image matching directly determine the results of visual positioning, so image matching has become the main topic of this study. In this study, the neural network algorithm is systematically optimized, especially for the robot's local obstacle avoidance, and an obstacle data acquisition method based on VGG16 and fast RCNN is proposed. In order to solve the problem that the semantic image segmentation algorithm based on AlexNet and ResNet is difficult to accurately obtain the information of multiple objects, and an image semantic segmentation algorithm combined with VGG16 is designed to classify the background and road in the image at the pixel level and capture the path boundary line. The collection of robot obstacle path information improves the speed and accuracy of highly automated local obstacle avoidance. This study uses neural network algorithms to systematically optimize computer vision positioning and also studies the accuracy optimization of local obstacle avoidance, aiming to promote its better development.

## 1. Introduction

Nowadays, life has become more and more convenient. Due to the development of society and the proliferation of smartphones and other devices, people's daily travel is completed through positioning and navigation. Therefore, location information has attracted more and more public attention, and the demand for location information has gradually increasedik. In the positioning system, the most important issue is whether the collection of location information is accurate and the real-time nature of the collection, which is a technology that must be considered in the positioning technology [1]. Now, in an open environment, GPS positioning signals can provide accurate locations of buildings, cars, and people and provide the best navigation route, which can basically meet people's normal needs [2]. However, for the indoor environment, the complexity of the environment is high, which leads to a sharp decline in GPS signals, and it is difficult to obtain positioning information in an indoor environment. However, the indoor visual positioning of the motion carrier provides a lot of support for the independent operation of the robot [3]. Therefore, the computer vision positioning system of the motion carrier is a suitable research field and a good research direction [4]. Then, this study also studies the local obstacle avoidance of robots based on neural network algorithms [5]. The research of mobile robots avoiding obstacles is a hot frontier in the field of mobile robot control. For road robots, autonomous part avoidance of sudden obstacles such as people is a prerequisite for effective road operations, which is an important guarantee for road robots to achieve autonomous, safe, and reliable operation [6]. This study focuses on the autonomous local obstacle avoidance method of robot dynamic obstacle path, obstacle information acquisition, and obstacle planning movement [7]. In terms of mobile robot data collection, semantic image segmentation algorithms can achieve pixel-level classification based on neural convection networks [8]. By dividing and labeling different areas of the image, it can accurately obtain obstacle information compared to other image information acquisition methods [9]. However, when using the semantic image segmentation algorithm based on AlexNet and ResNet to obtain dynamic obstacle path information, training is not easy, and it is difficult to accurately access multiple objects in a single segmentation area [10]. In terms of obstacle avoidance movement planning, compared with the artificial potential field method, probability graph method, and geometric optimization method, the speed obstacle method has better real-time performance and can eliminate the path jitter problem in the dynamic obstacle avoidance process [11]. Without considering the problem of the robot's own motion information, the accuracy of obstacle-climbing motion planning is low [12]. Therefore, this study studies a data acquisition method based on VGG16 and Fast RCNN to improve the speed of data acquisition and system optimization [13]. At the same time, road robots research the obstacle avoidance motion planning method based on the improved speed obstacle method, which improves the obstacle motion planning [14]. The accuracy also improves the speed of autonomous obstacle avoidance of road robots [15–18].

## 2. Related Work

The literature proposes a road sweeping robot obstacle avoidance motion planning method based on the improved speed obstacle method. In order to solve the problem of low accuracy when avoiding obstacles in the existing speed obstacle method without considering the robot's own motion information, it combines obstacle information to complete the speed obstacle method to improve the accuracy of the road sweeping robot to avoid obstacles [19]. The literature builds an experimental platform for the autonomous local obstacle avoidance system of a road sweeping robot [20–23]. The hardware platform is designed to complete the image collection and transmission of the road sweeping robot [24]. MATLAB is used to design and train the network model. The literature designs an image algorithm combined with VGG16 semantic segmentation to classify the sky, background, and roads in the road image at the pixel level and capture the road boundary, which can improve the convergence of the model. The literature proposes a road sweeping robot obstacle avoidance motion planning method based on the improved speed obstacle method [25]. By combining the obstacle information with the robot's own fast motion information, the speed obstacle method is improved, and the accuracy of avoiding autonomous local obstacles is improved [26]. The literature studies the self-positioning principle of computer vision, understands the basic principle and process of self-positioning and sensing motion of the carrier in computer vision, introduces its specific application steps in detail, and then develops an obstacle data collection method based on VGG16 and Faster RCNN to improve the convergence of the model that is obtained, and the information of multiple objects in the same segmentation area is obtained, thereby improving the speed and accuracy of obtaining obstacle information [27–30]. At the same time, based on the improved speed obstacle method, the robot avoiding obstacles in the sidewalk is studied. The accuracy of motion planning is improved, and therefore, the speed and accuracy of the robot's autonomous local obstacle avoidance are further improved [31–35].

## 3. Neural Network Algorithm and Computer Vision Positioning Model

### 3.1. Neural Network Algorithm

#### 3.1.1. Basic Algorithm

The output of the traditional neural network is completely based on the actual input, which makes the traditional neural network unable to make good predictions on the data that change over time. The output of the repetitive neural network is determined by the input at the current time and the output at the previous time [36, 37]. It is like adding memory space to the neural network so that the neural network can remember the behavior of the previous moment and respond to the current symptoms according to the behavior of the previous moment and the behavior at this time, as if the traditional neural network is disconnected, and the repeated neural network is not interrupted [38–40].

The recurrent neural network repeatedly calculates the weights at different times at the same time. When the time series are discovered, the past state is theoretically transferred to the present, which means that today's results are not only affected by today's events but also related to yesterday. This calculation method based on the recurrent neural network has been widely used in speech recognition, natural language processing, stock evaluation, and other issues [41–43].

The forward propagation process of RNN is as follows:

(1) $$\begin{array}{c} o_{t}=g{\left(V_{L\times M}\cdot h_{t}+by_{L\times 1}\right)}_{L\times 1}, \end{array}$$

(2) $$\begin{array}{c} h_{t}=f{\left(U_{M\times N}\cdot x_{t}+W_{M\times M}\cdot h_{t-1}+bh_{M\times 1}\right)}_{M\times 1}. \end{array}$$

The objective function is set to the following:

(3) $$\begin{array}{c} E=\sum_{t=1}^{T}E_{t}\\ =\frac{1}{2}\sum_{t=1}^{T}\sum_{l=1}^{L}{\left(y_{lt}-o_{lt}\right)}^{2}. \end{array}$$

#### 3.1.2. Model Improvements

In order to make the RNN algorithm converge faster, this chapter introduces the random perturbation parameter *μ*M×*N* of the hidden layer of the RNN algorithm and adds it to the weight of the input layer to obtain a new weight. The forward propagation process of the RNN enhanced network is as follows:

(4) $$\begin{array}{c} {\dot{U}}_{M\times N}=U_{M\times N}+\mu_{M\times N}, \end{array}$$

(5) $$\begin{array}{c} {\left(h_{t}\right)}_{M\times 1}=f\left({\dot{U}}_{M\times N}\cdot {\left(x_{t}\right)}_{N\times 1}+W_{M\times M}\cdot {\left(h_{t-1}\right)}_{M\times 1}+{\left(bh\right)}_{M\times 1}\right), \end{array}$$

(6) $$\begin{array}{c} {\left(o_{t}\right)}_{L\times 1}=g\left(V_{L\times M}\cdot {\left(h_{t}\right)}_{M\times 1}+{\left(by\right)}_{L\times 1}\right). \end{array}$$

The objective function is selected as mean squared error (mean squared error):

(7) $$\begin{array}{c} E^{ps}=\frac{1}{2}\sum_{j=1}^{J}\sum_{t=1}^{T}\sum_{l=1}^{L}{\left(y_{tl}^{sj}-o_{tl}^{sj}\right)}^{2}. \end{array}$$

In this study, a total of 1,251 one-dimensional data are selected, the first 1,000 items are used as the training data set, and the remaining 251 items are used as the prediction set data. The RNN algorithm constructed by NumPy of Python software adds random interrupts during the forward motion. The standard evaluation of the experiment is to test the loss performance of the system. Compared with the RNN before the improvement, the random perturbation of the current RNN significantly improves the convergence speed [44, 45].

Compared with the previous RNN, the improved RNN has a more stable convergence, with less loss, and a better convergence effect than the original one.

The comparison of some losses after the convergence is stable and is shown in Table 1:

Table 1 shows the RNN prediction result table, in which the training has exceeded 1,600 times, and the loss before improvement will not exceed 0.02, nor will it be lower than 0.009, and the loss after improvement will not be higher than 0.018, and it will not be lower than 0.007.

### 3.2. Computer Vision Positioning Technology

In search machine vision, the position of the moving carrier image is realized according to the sequence relationship between similar mining images. For example, cameras and cameras are widely used. They can provide a wealth of image data information for the visual positioning system and provide a good foundation for the study of the visual self-positioning of the motion carrier. In addition, visual sensor calibration is easy, image matching and other algorithms are more frequently searched, and the application is also widely used, which provides a good platform for the research of this subject. The steps of the indoor sports carrier visual positioning test are as follows:

1. A motion carrier with a vision sensor is used to collect image information of an indoor scene, and images of the same scene are collected. Every time, the pictures are taken clockwise at 30° intervals, and the coordinates of the collected images and the camera rotation angle are recorded.
2. The collected images are processed, the global features and local features are sorted, and then, feature descriptors are created based on the feature description algorithm.
3. The detailed description of the collected data images corresponds and saves the coordinates and angles of the corresponding motion carriers and builds an environmental feature library.
4. Fifty visual images are randomly taken, and the coordinates and angles are recorded as test images. Image processing is performed on the test image, similar image information is extracted from the feature library, and compatible coordinates with it are found.
5. According to the geometric principle, the corresponding straight line between the matching images is solved.
6. For the extracted data image coordinates and camera parameters, the coordinates of the image position are calculated.

**Figure 1:**
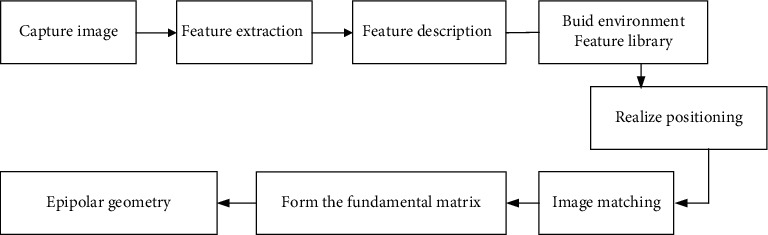
The flowchart of the visual positioning algorithm of the motion carrier.

According to the internal and external parameters obtained after the camera calibration, the experimental photographs were tested and the coordinates of the moving carrier were determined. The specific flowchart is shown in Figure 1.

According to the flowchart in Figure 1, the specific scheme of the experiment is obtained. First, the selection of experimental scenes and the collection of experimental images establish an environmental feature library. The establishment of the environmental feature library requires a standard coordinate system to capture visual images and perform feature extraction steps, in order to facilitate the collection of experimental images and subsequent image processing and matching experiments. In this experimental stage, there are some changing factors, enclosed objects, etc., which will affect the experimental results of the indoor positioning system. Therefore, the indoor stage corresponding to the actual position can be used as the experimental system test stage.

Image processing is performed on the data images collected in the experimental scene, the image features are described according to the algorithm, digital information is generated, the environment feature library is established, and the current coordinate positions of the images recorded during the experience are stored. Generally speaking, the images collected by the vision sensor are divided into two types: reference points and video streams. Based on the video stream method, one-frame image or multiple frame images are extracted from the experimental video shooting scene, which is used to extract features for experimental matching, and the coordinate position of the collected image is calculated by formula (8).

(8) $$\begin{array}{c} \left\{\begin{array}{c} X_{wn}=X_{w0}+v\frac{m}{m}\cos\;\alpha ,\\ Y_{wn}=Y_{w0}+v\frac{n}{m}\sin\;\alpha . \end{array}\right. \end{array}$$

## 4. Optimization of Local Obstacle Avoidance Algorithm and System Design

### 4.1. Local Obstacle Avoidance System Architecture

The road motion planning of the acceleration robot is divided into two types according to the environmental information in the acquisition area: a global motion plan and a plan to avoid local obstacles. Among them, planning a global movement first needs to obtain complete environmental information in the area, and when obtaining global environmental information, a route from the starting point to the target point should be planned. Global motion planning can comprehensively analyze global environmental information to find the best path. But in the absence of overall information or emergency situations, the action plan does not work very well. The local obstacle avoidance movement plan is to update the original obstacle avoidance method according to the obstacle information discovered in real time by the camera and other sensors when the road sweeping robot is in a local unknown environment. The local movement plan that avoids obstacles can cope with real-time emergencies, but the level of sensor detection of obstacle information is limited, and it is easy to fall into a local optimal solution. Its real-time nature ensures that the task of avoiding performance obstacles can be properly completed.

This study focuses on the autonomous system of road sweeping robots avoiding local obstacles. The system consists of the following three parts:

1. Road environment modeling. For the locally autonomous method of avoiding obstacles in the sweeping robot, the road environment should be premodeled based on the information obtained from the sensors. The purpose is to convert road environmental information into data that the controller can identify and analyze, form a road ecological model, and convert road environmental information into data so that the controller can analyze environmental information.
2. Search for local routes. The best-planned route is found according to the environmental information parameters of the road sweeping robot controller. The local path research is the most critical part of the robot's local autonomous obstacle avoidance method, which is closely related to the road ecological model in the previous stage. It can not only plan a specific route but also plan some parameters such as the direction to avoid obstacles or the speed to avoid obstacles.
3. Execute partial routes. According to the results of the local path search, the corresponding number of the controller is provided under the road sweeping robot controller so that the road sweeping robot can move as expected. The realization of the local path is directly related to the degree of automation of the floor controller of the sweeping robot, and the operability and controllability of the sweeping robot.

In the above three parts, the basic content of the local autonomous method of robot avoiding obstacles is the road environment model and local path research. Obstacle information acquisition in this study corresponds to the two steps of the obstacle avoidance movement plan.

Real-time measurement and control of multisensor parameters and accurate implementation of algorithms for avoiding local obstacles are essential for fast road robots to avoid autonomous local obstacles. Therefore, when designing the hardware composition of the robot path, it is necessary to create a reliable and effective automatic sweep path autonomous local obstacle avoidance system.

The airborne sensor decision-making module is composed of the following 5 submodules:

1. Module location. During the cleaning operation, the road sweeping robot must not only be able to autonomously avoid local obstacles in an emergency but also must be able to return to the originally planned global route after overcoming the obstacles. Therefore, it is very important for a road sweeping robot to avoid local obstacles and accurately retrieve its own posture information in real time. The basic navigation and positioning module requires the global positioning system to find the latitude and longitude of the road sweeping robot, and the GPS obtains the position information and movement information of the road sweeping robot.
2. Environmental awareness module. Local autonomous systems rely on sensors to obtain environmental information, quickly avoid obstacles on the road, and have high requirements for real time and reliability of road environment models that include various environmental information parameters. The sweeping robot in this study uses a camera to capture road images in real time and uses conventional neural networks to process the images to obtain road and obstacle information.
3. Power module. In this study, the power supply of the sweeping robot is 24V, and its power module is composed of a high-performance lithium iron hydrochloride battery with a discharge capacity of 30Ah and a stable circuit voltage.
4. Drive module. The driving module of the sweeping robot in this study uses 4 single-axis intelligent motor modules, which can drive the robot to move forward, backward, and rotate to ensure that it can also perform cleaning operations in a narrow road environment.
5. Communication module. The sweeping robot communication module consists of two parts: a wireless router and a radio station. The wireless router used to transmit image information is captured by a camera with a large amount of data, while the radio station is used to transmit the amount of data and information to the robot sweeping path, and relatively small motion state information is output to the control terminal through the data analysis module.

In addition to building the system hardware platform, the system must also meet the robot's autonomous local obstacle avoidance, that is, avoiding the robot's local obstacles in cleaning the road. It consists of the following three parts:

1. Human-computer interaction module. In order to facilitate the work of the monitor at the monitoring end, the human-computer interaction interface in the upper computer at the control end is designed to provide a flexible window end and monitoring end information analysis system. The human-computer interaction module is bidirectional and can receive the information transmitted by the sensor decision module so that the monitor can understand the real-time information of the robot cleaning path and at the same time send the cleaning robot path to the robot to achieve human control of the robot.
2. Environmental awareness module. In order for the road cleaning robot to meet the requirements of obstacle information collection, the environment scene module must find the road environment information according to the image taken by the camera and then construct the environment model of the road cleaning robot based on the environment information. For the environment perception module, the road image taken by the camera is abstract, so it is necessary to use the image semantic segmentation algorithm to process the road image to obtain obstacle information, and to identify and analyze the image system data through the converted data.
3. Movement planning module. The environment perception module receives the obstacle information and transmits it to the motion planning module and plans an appropriate way to avoid the obstacle in the known local environment. When the environment perception module detects an obstacle, the robot replans the path according to the rules and changes some path nodes to avoid the obstacle.

### 4.2. Optimization Model of Local Obstacle Avoidance Algorithm

Semantic segmentation algorithms usually analyze and explore image shape and edge information. The complexity of the algorithm and the difficulty of convergence make the entire semantic segmentation process very time-consuming. Compared with traditional image semantic segmentation algorithms based on regional features, neural convection networks are smaller and more efficient and do not need to search for target image regions and fusion activities. Through convolution and pooling operations, the feature map is reduced, and the acquired image features are redrawn into the image.

The neural convolutional network structure usually consists of the following: convolutional layer, pooling layer, and fully connected layer. The convolutional layer is the basic part of the neural convolutional network. It can filter a specific area of the image to obtain a feature map and then use a nonlinear activation function to perform nonmapping operations. Nonlinear activation functions include linear rectification functions, s-curve functions, and tan-curve functions.

The formulas are (9) to (11).

(9) $$\begin{array}{c} f\left(x\right)=\max\left(0,x\right), \end{array}$$

(10) $$\begin{array}{c} f\left(x\right)=\frac{1}{1+e^{-x}}, \end{array}$$

(11) $$\begin{array}{c} f\left(x\right)=\tanh\left(x\right)=\frac{e^{x}-e^{-x}}{e^{x}+e^{-x}}. \end{array}$$

Compared with the sigmoid and tanh functions, the ReLU function has no exponential calculation. Using thresholds to calculate nonlinear activation values can increase the speed of model convergence and model training.

After convolution, the picture is pooled. The general principle of the pooling layer is as follows:

(12) $$\begin{array}{c} y_{i,j,k}^{l}=\text{pool}\left(a_{m,n,k}^{l}\right),\forall \left(m,n\right)\in R_{i,j}. \end{array}$$

This pooling has two main functions: compressing data and parameter values, which can be operated to reduce fitting, thereby improving the model's rotation change and fault tolerance. After conversion and pooling, the conventional neural network mapping and data lead to the fully connected layer. Each node of the fully connected layer of the neural convolutional network is connected to all the nodes of the previous layer to combine the captured features.

In the neural multilayer convolutional network, the encoder mainly captures the image features of the convolutional layer and the pooling layer. The encoder can extract local features of the image through architectures such as AlexNet, VGGNet, and ResNet.

The goal of semantic image segmentation is to divide image regions into different categories, and each image pixel must be identified and sorted. The segmented image has the same size as the original image, and the image size of the encoder layer is reduced by layer, and then decoded layer by layer to increase, so as to restore the feature map and obtain an image with the same size as the original image.

Every regular layer in the encoder is capturing image attributes. In order to minimize the unnecessary information in the image, the pooling layer after each group of convolutional layers adopts maximum pooling and records the index to facilitate the use of the decoder. According to the maximum pooling index, 0 is added to the blank area, and you can gradually enlarge it according to the zoom ratio analyzed by the two pooling layers in front of each conventional layer group in the decoder and finally restore the original image size to find a sparse response map. Finally, softmax is used to obtain the classification of individual pixels in the image.

According to whether or not to set up manually labeled image training, CNN can be divided into supervised exercises and unsupervised exercises. This study uses supervised CNN to train the image segmentation model.

The supervised learning process involves training with manually labeled datasets, CNN, to obtain functions that meet the requirements of the training series and testing the CNN using test data that has not been manually identified. The training effect is confirmed according to the accuracy of the test sequence. In supervised practice, stochastic gradient descent is the simplest and most effective method, which is mainly used to learn linear classification under convex loss functions, such as support-vector machines and logistic regression. SGD error function principle is as follows:

(13) $$\begin{array}{c} J\left(W,b;x,y\right)=\frac{1}{2}\sum_{k=1}^{N}{\left(t_{k}-y_{k}\right)}^{2}=\frac{1}{2}{\left\|t-y\right\|}_{2}^{2}. \end{array}$$

In the backpropagation process, the output layer derives the neural network error value of each layer and calculates the error value affected by each connected layer. If the *L* layer and the *l*+1 layer are fully connected to the network model, the error value Ϭ(l1)+ of the *l*+1 layer can be derived from the error value Ϭ(l) of the *l* layer. The calculation formula is as follows:

(14) $$\begin{array}{c} \delta^{\left(1\right)}=\left({\left(W^{\left(1\right)}\right)}^{T}\delta^{\left(1+1\right)}\right)\cdot f^{\prime }\left(z^{\left(1\right)}\right). \end{array}$$

The gradient formula for W^(l)^ and b^(l)^ can be calculated as follows:

(15) $$\begin{array}{c} \nabla_{W^{\left(1\right)}J\left(W,b;x,y\right)}=\delta^{\left(1+1\right)}{\left(f^{\prime }\left(z^{\left(1\right)}\right)\right)}^{T}. \end{array}$$

(16) $$\begin{array}{c} \nabla_{b^{\left(1\right)}}J\left(W,b;x,y\right)=\delta^{\left(1+1\right)}. \end{array}$$

If the lth layer in the network model is a convolutional layer, the error value formula is as follows:

(17) $$\begin{array}{c} \delta_{k}^{\left(1\right)}=\text{ unsample }\left({\left(W_{k}^{\left(1\right)}\right)}^{T}\delta_{k}^{\left(1+1\right)}\cdot f^{\prime }\left(z^{\left(1\right)}\right)\right). \end{array}$$

In this case, the gradient calculation is performed, and the process can be expressed as follows:

(18) $$\begin{array}{c} \nabla_{w_{k}^{\left(1\right)}J\left(W,\;b;x,y\right)}=\sum_{i=1}^{m}a_{i}^{\left(1\right)}*rot90\left(\delta_{k}^{\left(1+1\right)},2\right), \end{array}$$

(19) $$\begin{array}{c} \nabla_{b_{k}^{\left(1\right)}}J\left(W,b;x,y\right)=\sum_{a,b}{\left(\delta_{k}^{\left(1+1\right)}\right)}_{a,b}. \end{array}$$

After completing the backpropagation, the gradient descent method needs to be used to update the weights. The calculation formula is as follows:

(20) $$\begin{array}{c} \theta =\theta -\partial \nabla_{\theta }J\left(\theta ,x,y\right). \end{array}$$

The semantic image segmentation model mainly considers three factors: pixel accuracy, area intersection, and parallelism. There are four evaluation indicators: pixel accuracy, class average accuracy, average intersection, and parallel ratio. The intersection ratio combines model estimation and manual annotation, that is, the intersection ratio between model prediction results and manual annotation results. The formula is as follows:

(21) $$\begin{array}{c} IOU=\frac{DR\cap GT}{DR\;UGT}. \end{array}$$

Pixel accuracy is the proportion of correctly classified pixels in the total pixels. The calculation formula is as follows:

(22) $$\begin{array}{c} \frac{\sum n_{i,i}}{\sum t_{i}}t_{i}. \end{array}$$

Class average accuracy is the average classification accuracy of each category, and the calculation formula is as follows:

(23) $$\begin{array}{c} \frac{1}{n_{c,1}}\sum_{i}\frac{n_{i,i}}{t_{i}}. \end{array}$$

The average intersection is the average degree of various types of IoU, and the calculation formula is as follows:

(24) $$\begin{array}{c} \frac{1}{n_{c_{r}l}}\sum_{i}\frac{n_{i,i}}{\left(t_{i}+\sum_{i}n_{j,i}-n_{i,i}\right)}. \end{array}$$

The weighted intersection ratio is the average degree of IoU in different categories. The weight value is the pixel ratio of a category. The calculation principle is as follows:

(25) $$\begin{array}{c} {\left(\sum_{i}t_{i}\right)}^{-1}\cdot \sum_{i}t_{i}\frac{n_{i,i}}{\left(t_{i}+\sum_{i}n_{j,i}-n_{i,i}\right)}. \end{array}$$

**Figure 2:**
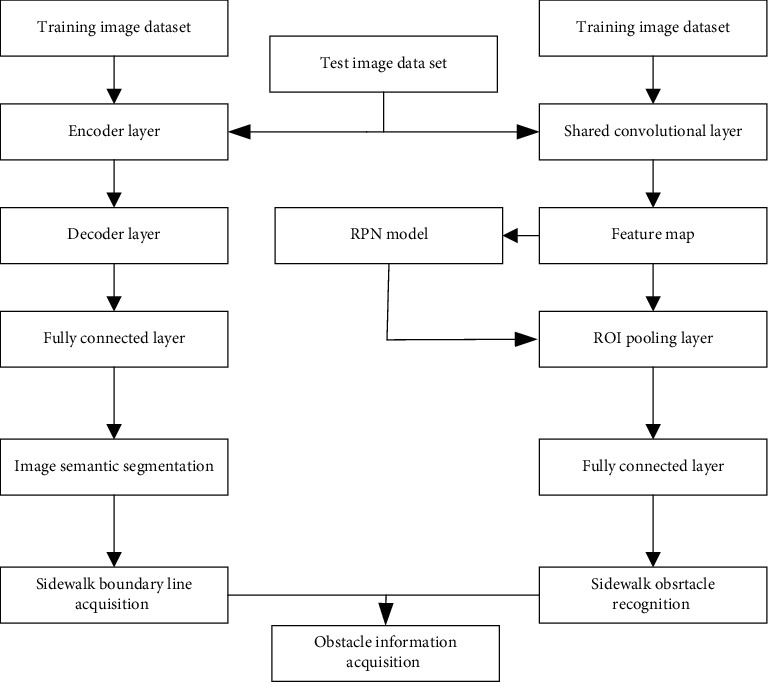
A framework method of Fast RCNN obstacle data acquisition based on VGG16 for robot sweeping.

Figure 2 shows the basic information knowledge of the road sweeping robot obstacle data collection method based on VGG16 and Fast RCNN.

The method steps are as follows:

1. Image data series are prepared for training semantic segmentation models and Faster RCNN models. The semantic image segmentation model data and the Faster RCNN model require road images and corresponding pixel-level calibration results and calibration targets, respectively;
2. The training images set on the VGG16 and Fast RCNN models are input until the bias value and weight distribution of the network model are satisfied and stored;
3. The test set image is input into the trained model of VGG16, across the coding layer, decoding layer, and fully connected layer, and then, the semantic segmentation result of the test image is output.
4. The test set image on the fast model trained by RCNN is input, and the test set image is passed on the convolutional layer, RPN network, ROI pool layer, and fully connected layer. The target detection result is obtained, and then, the obstacles in the input image are classified according to the pedestrian category and other obstacles. The intersection ratio between the road area output is calculated by the VGG16 model and the prediction box output by the fast RCNN model, and then, the test result is invalidated;
5. According to the principle of camera homography, the interaction between the world coordinate system and the pixel coordinate system realized by the external parameters is reflected by the camera installation position and the internal parameters obtained by the camera calibration. Through the current edge coordinate value of the road boundary line and the coordinate value of the bottom edge line of the target estimation frame, the result of the obstacle data collection of the fast road robot based on the convolutional network is output.

The VGG16 model can achieve the accurate pixel-level classification of different areas such as the sky, background, and roads and complete the acquisition of road edge lines; RCNN's fast network model can accurately identify and classify roadblocks so that the road sweeping robot can take appropriate measures against various obstacles. Avoidance measures, combining the boundary line recognition results and the obstacle path recognition results, can get the final image advance collection result, which provides the information needed by the environment for the next move planning method.

### 4.3. Model Simulation Experiment Results

In the recognition process, the time consumed per frame is between 0.123s and 0.159s, and the average time is 0.1388s frame. It can be seen that the trained model takes less time to detect the test range.

The same training set is used to design AlexNet and ResNet models, and the trained model is used to identify in the same test to get the average time and accuracy of data acquisition in each algorithm. The comparison information is shown in Table 2.

The same dataset is used to apply the obstacle avoidance motion planning method to improve the obstacle speed method, the obstacle avoidance result is compared with the obstacle method result to be applied by the system, and the obstacle avoidance accuracy is calculated. The accuracy of the two methods is shown in Table 3.

The experimental results show that the accuracy of the obstacle avoidance movement plan based on the obstacle speed improved speed obstacle method is 5.1% higher than that of the nonimproved speed obstacle method.

**Figure 3:**
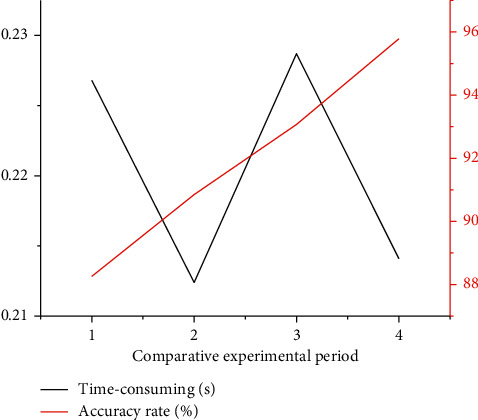
Comparative experimental results of autonomous local obstacle avoidance.

By combining the above experiments, the results of the four groups of autonomous robots avoiding local obstacles are compared, and the extreme situation of poor obstacle information acquisition is not considered. The results are shown in Table 4 and Figure 3.

Based on AlexNet, ResNet, and the existing speed obstacle method, the autonomous local obstacle avoidance method using the proposed dynamic obstacle route reduces the obstacle avoidance time by 5.68% and the obstacle accuracy rate increases by 7.52%. The experiment proved the effectiveness and advantages of the local autonomous obstacle avoidance method.

As the test site of this study, the road width is between 2 m and 2.5 m, which is 0.25 m higher than the roadway. The experiment also recorded the time taken for the robot to avoid obstacles and the minimum distance between the robot and pedestrians in the case of 4 kinds of obstacles. The experimental results are shown in Table 5.

Experimental results show that the robot can correctly avoid obstacles under four conditions: avoiding obstacles and maintaining the minimum safe distance of more than one fuselage. During the operation of the robot, the total angular velocity changes within a small range. Within the controllable range, the path has no jitter phenomenon, the obstacle avoidance trajectory is smooth, and the stability is good. It is proved that the proposed autonomous local obstacle avoidance system route can accurately identify obstacles and formulate a fast and accurate obstacle avoidance plan.

## 5. Conclusions

With the rapid development of positioning technology, people basically have positioning software on their mobile phones. At present, the positioning technology for outdoor environments is very mature, and outdoor positioning is mainly based on GPS, satellites, etc. But for the positioning system in the indoor environment, there is no mature technology that can be completed at present. GPS and satellites that rely on outdoor positioning are greatly affected by the occlusion of indoor walls, and the positioning effect is not so good, so they are not suitable for indoor positioning systems. Indoor WiFi systems are also limited by space and need to frequently update the database, and the update cost is not low. When building an indoor positioning system with sensors such as lasers, it is also difficult to establish a database of environmental characteristics. The main reason for choosing visual positioning is that the development of visual sensors is great, the image information captured is also rich, and the equipment is simple and convenient. Therefore, this study studies a Faster RCNN data collection method based on VGG16 to improve the speed and accuracy of data collection. At the same time, it also studies the road robot obstacle avoidance method based on the improved obstacle method, which improves the road robot obstacle avoidance motion planning. The research content of this study is mainly divided into two parts: neural network algorithm and computer vision positioning model, local obstacle avoidance algorithm optimization, and system design. The model mainly involves neural network algorithm and computer vision positioning technology, while algorithm optimization and system design mainly include local obstacle avoidance system architecture, local obstacle avoidance algorithm optimization model, and model simulation experimental results.

## Figures and Tables

**Table 1:** RNN prediction results.

Training times	Loss before improvement	Improved loss
121	0.01846273	0.01644995
122	0.01649239	0.01440386
123	0.01985817	0.01450079
124	0.01938425	0.01751673
125	0.01606731	0.01726111
126	0.01539695	0.01439848
127	0.01632119	0.01343476
...	...	...
1725	0.01102587	0.00894695
1726	0.00987087	0.00806328
1727	0.00984348	0.00812178
1728	0.01192545	0.00971999
1729	0.01210827	0.01016545
1730	0.00972976	0.00813577
1731	0.00934806	0.00766848

**Table 2:** Comparison of various algorithms.

Algorithm	Training process time (min)	Average time for information acquisition (s)	Accuracy rate (%)
AlexNet	222	0.1611	91.91
ResNet	171	0.1534	94.38
VGG16+Faster RCNN	134	0.1388	97.15

**Table 3:** Comparison of the accuracy of obstacle avoidance movement planning.

Algorithm	Obstacle avoidance success rate (%)
Existing speed obstacle method	93.52
Improved speed obstacle method	98.62

**Table 4:** Comparative experimental results of autonomous local obstacle avoidance.

Obstacle information acquisition method	Obstacle avoidance movement planning method	Time-consuming (s)	Accuracy rate (%)
AlexNet/ResNet	Existing speed obstacle method	0.2268	88.26
VGG16+Faster RCNN	Existing speed obstacle method	0.2124	90.85
AlexNet/ResNet	Improved speed obstacle method	0.2287	93.07
VGG16+Faster RCNN	Improved speed obstacle method	0.2141	95.78

**Table 5:** Robot obstacle avoidance experiment.

Obstacle situation	Average time (S)	Minimum distance (m)
Chase over	15.47	2
Encounter	5.14	0.9
Right cross	8.32	1.2
Jump in place	6.83	1.1
Crawl	7.95	1.3
Left cross	8.28	1.2

## Data Availability

The datasets used and/or analyzed during the current study are available from the corresponding author on reasonable request.
